# The Deadly Toy Pistol

**Published:** 1906-02

**Authors:** 


### The Deadly Toy Pistol.

Immediately after Christmas and in the early part of January the daily newspapers teemed with accounts of accidents from the use of fireworks during the holidays. Many fatalities were reported and in a very large number of instances death resulted from lockjaw brought on by wounds from common toy pistols. As the practice of using fireworks for Christmas is more general in the South than in the North, a large percentage of the fatal accidents occurred in Southern cities and towns.

The dangerous character of wounds inflicted by toy pistols has been frequently pointed out and medical practitioners have advanced learned theories as to why lockjaw so frequently follows; many cities and towns have passed ordinances restricting or prohibiting the sale and use of these toys which seem to be particularly destructive to human life. Such ordinances, however, are usually passed immediately after the celebration has left its train of woe and sorrow, and once enacted, they seem generally to pass at once into oblivion; being remembered no more by officers or people until after another celebration brings its quota of disasters. It is the old story of locking the door after the horse is gone—only, unfortunately, the door isn't kept locked; it is wide open on the next occasion when locks and bars are needed.

Officers, like others, are inclined to overlook minor infractions of the law in times of general rejoicing or celebration. No one desires to curtail the enjoyment of the children at any time and above all at the Christmas season. But is it kindness to give them freedom to injure themselves and others? Is the ecstacy found by boyish ears in listening to the "pop-pop" of the toy pistol worth the hours of agony, the cutting off of innocent lives which it yearly entails? Loving parents who place flowers on freshly turned little mounds perhaps do not think so.—*Holland's Magazine.*

